# Supplementary material for: Cost-Effectiveness of Bivalent Respiratory Syncytial Virus Prefusion F Vaccine for Prevention of Respiratory Syncytial Virus Among Older Adults in Greece
Source: Vaccines (Basel). 2024 Oct 29;12(11):1232. doi: 10.3390/vaccines12111232 (PMC11598676; doi:10.3390/vaccines12111232)
Supplement: Supplementary file 1 [file vaccines-12-01232-s001.zip › vaccines-3225668-supplementary.pdf]

Supplementary Material

Table S1. Probabilistic sensitivity analysis variables sampled and distributional information.

Variable	Distribution	Parameters
RSV Rates (annual, per 100K)		
Hospitalized	Beta	Alpha: 2,140; Beta: 2,285,378
Case-Fatality Rate (≤30 days)		
Hospitalized	Beta	Alpha: 8,963; Beta: 112,568
Vaccine Effectiveness		
Hospitalized	Beta	Alpha: 6.8; Beta: 1.2
Outpatient	Beta	Alpha: 7.8; Beta: 3.7
Gen. Pop. Utilities	Beta	Alpha: 63,818,418; Beta: 5,589,398
Disutilities (QALY loss)		
Hospitalized	Beta	Alpha: 4.0; Beta: 234.0
Outpatient	Beta	Alpha: 19.5; Beta: 3,580.7
Direct Medical Care Costs (per case)		
Hospitalized	Log-Normal	Mean: 4,214; SE: 65.98
Outpatient	Log-Normal	Mean: 283; SE: 70.75
